# Mir-30b-5p Promotes Proliferation, Migration, and Invasion of Breast Cancer Cells via Targeting ASPP2

**DOI:** 10.1155/2020/7907269

**Published:** 2020-04-29

**Authors:** Tianqi Wu, Hongming Song, Dan Xie, Kaiyao Hua, Jiashu Hu, Yijun Deng, Changle Ji, Lin Fang

**Affiliations:** ^1^Department of Breast and Thyroid Surgery, The Affiliated Huaian Hospital of Xuzhou Medical University, Huaian 223000, China; ^2^Department of Breast and Thyroid Surgery, Shanghai Tenth People's Hospital, Tongji University School of Medicine, Shanghai 200072, China; ^3^Breast Disease Center, The Affiliated Hospital of Qingdao University, Qingdao 266000, Shandong, China

## Abstract

Triple-negative breast cancer (TNBC) is the most aggressive subtypes of breast cancer, which has few effective targeted therapies. Various sources of evidence confirm that microRNAs (miRNAs) contribute to the progression and metastasis of human breast cancer. However, the molecular mechanisms underlying the changes in miRNAs expression and the regulation of miRNAs functions have not been well clarified. In this study, we found that the expression of miR-30b-5p was upregulated in breast cancer tissues and breast cancer cell lines, compared to paracancer tissues and normal breast cell lines. Moreover, induced overexpression of miR-30b-5p promoted the MDA-MB-231 and HCC 1937 cell growth, migration, and invasion and reduced the cellular apoptosis. Further studies confirmed that miR-30b-5p could directly target ASPP2 and then activate the AKT signaling pathway. Our results suggested that miR-30b-5p could act as a tumor promoter in TNBC. The newly identified miR-30b-5p/ASPP2/AKT axis represents a novel therapeutic strategy for treating TNBC.

## 1. Introduction

Breast cancer is one of the most prevalent causes of cancer-related death among females worldwide, with morbidity and mortality becoming younger in average gradually [1,2]. Triple-negative breast cancer (TNBC), which accounts for approximately 12–20% of all breast cancers [3], is characterized by a high rate of proliferation and invasion, as well as rapidity of metastatic formation [4]. Few effective therapies are available for TNBC until now; this is because TNBC patients lack estrogen receptors, progesterone receptors, and human epidermal growth factor receptor 2 (HER2) [5]. Surgical resection and chemotherapy are the main treatments for TNBC, but the postoperative recurrence and chemotherapy resistance always result in the treatment failure [6]. Therefore, biomarkers for TNBC are urgently needed to find novel targeted therapeutic strategies.

MicroRNAs (miRNAs) are a class of small non-coding RNAs that are 19–25 nucleotides in length, which modulate gene expression by binding to the 3′-untranslated region (UTR) or, less commonly, the 5′-untranslated region (UTR) of the target messenger RNA (mRNA) [7,8]. Emerging literature suggests miRNAs may function either as oncogenes or tumor suppressor genes. It has been identified that miRNAs play an important role in biological processes of breast cancer, such as cell proliferation, cell apoptosis, invasion, and resistance to therapy [9–11].

Recently, miR-30b-5p, a member of miR-30b family, was found to be associated with the development of many types of cancers. However, the role of miR-30b-5p is intricate, even controversial. For instance, miR-30b-5p functions as an oncogene in Medulloblastoma [12] and malignant mesothelioma [13], but acts as a tumor suppressor in prostate cancer [14], bladder cancer [15], and gastric cancer [16]. It reveals a tissue type-dependent manner; that is, miR-30b-5p may play different roles in different cancers. However, the potential functions and mechanisms in TNBC are still unknown.

In this study, we found that miR-30b-5p was upregulated both in breast cancer tissues and breast cancer cell lines. Upregulation of miR-30b-5p promoted TNBC MDA-MB-231 and HCC 1937 cell proliferation, migration, and invasion but inhibited cell apoptosis. In addition, miR-30b-5p seemed to target ASPP2, thereby activating AKT signaling pathway in TNBC cells. Our results indicate that miR-30b-5p may function as an oncogene during the development of TNBC, which may provide a novel biomarker for diagnosis and therapy for TNBC.

## 2. Materials and Methods

### 2.1. Patients and Tissue Samples

14 paired human breast tumor samples and their matched adjacent nontumor tissues were collected from the Department of Breast and Thyroid Surgery of the Shanghai Tenth People's Hospital. The patients were women between the ages of 32 and 71 years, with a mean age of 56 years. Tissue samples were immediately sap-frozen in liquid nitrogen until total RNAs were extracted after surgical removal. All patients participating in the study received neither radiation therapy nor chemotherapy prior to surgery. All subjects gave their informed consent for inclusion before they participated in the study. The study was conducted in accordance with the Declaration of Helsinki, and the protocol was approved by the Ethics Committee of hospital (the approval number: SHSY-IEC-KY-4.0/17-49/01). The clinicopathologic information of the patients is summarized in Supplement Table I.

### 2.2. Cell Culture

Human breast cancer cell lines (MDA-MB-231, MCF-7, MDA-MB-468, and HCC 1937) and normal breast cells (MCF-10A) were purchased from Chinese Academy of Sciences in Shanghai. Cells were cultured in Dulbecco's Modified Eagle's Medium (DMEM; Gibco, Carlsbad, CA, USA) supplemented with 10% fetal bovine serum (FBS; Gibco, Carlsbad, CA, USA) and 1% penicillin⁄streptomycin (Gibco, Carlsbad, CA, USA). In addition, all of the cell lines were incubated at 37°C in a humidified atmosphere of 5% CO_2_.

### 2.3. Cell Transfection

For cell transfection, MDA-MB-231 cells (9 × 10^4^ per well) and HCC 1937 cells (1.5 × 10^5^ per well) were added into six-well plates respectively. When the cells were at 30–50% confluency, they were transfected with miR-30b-5p mimics and negative control (NC mimics), mir-30b-5p inhibitor and inhibitor negative control (NC inhibitor), which were synthesized and obtained by RiboBio (Guangzhou RiboBio Co, Guangzhou, China). Lipofectamine 2000 reagent (Thermo Fisher Scientific) was used to perform transfection according to the manufacturer's protocol. The transfected cells were cultured with serum-free DMEM for 6 h; the then medium was replaced with DMEM with 10% FBS. 48 h after transfection, the cells were harvested for following assays.

### 2.4. MTT Assay

For the 3-(4, 5-dimethylthiazol-2-yl)-2, 5-diphenyltetrazolium bromide (MTT) assay, cells were seeded into 96-well plates at a density of 500 cells per well. In order to quantitate cell viability, at different time points (24, 48, 72, 96, and 120 h), 20 *μ*l MTT solution (5 mg/mL; Sigma, St Louis, MO, USA) was added to each well and cells were incubated at 37°C for another 4 h. Then the medium was removed by aspiration, and 150 *μ*l dimethyl sulfoxide (DMSO; Sigma-Aldrich) was added into each well. After shaking for a further 10 min at a room temperature, absorbance value was measured at optical density (OD) of 490 nm using a microplate reader (BioTek, Winooski, VT, USA). Each experiment was performed independently in triplicate.

### 2.5. Colony Formation Assay

For colony formation assay, MDA-MB-231 and HCC 1937 cells were seeded into six-well plates at 1000 cells per well and incubated at 37°C incubator for 10 days posttransfection. Then the plates were washed with PBS, fixed with 95% ethanol for 15 min, and stained with 0.1% crystal violet for 20 minutes. Colony number was manually counted. Each experiment was performed at least 3 times.

### 2.6. Cell Apoptosis Assay

For measurement of cell apoptosis, Annexin V-FITC Apoptosis Detection kit (BD Biosciences, San Diego, CA, USA) was used following the manufacturer's instructions. After 24 h transfection, cells were treated with 1 *μ*mol/l Docetaxel for 36 h to induce apoptosis. Then cells were harvested and double-stained with fluorescein isothiocyanate (FITC)-conjugated Annexin V and propidium iodide (FITC-Annexin V/PI). The analysis of cell apoptosis were performed by flow cytometry (FACSCantoTM II, BD Biosciences), and the data were analyzed using CellQuest software (BD Biosciences).

### 2.7. Wound Healing Assay

To determine cell migration, transfected cells were seeded into 6-well plates and incubated until almost confluent. Subsequently, artificial wounds were created in the cell monolayer with a sterile 200 *μ*l tip, and the floating cells were removed by washing with PBS and incubated in serum-free DMEM to avoid cell proliferation. Respective images were recorded and captured at 0 and 48 h from the same position using an inverted microscope. Migration rate = (1 − distance at other time points/distance at 0 h) × 100%.

### 2.8. Transwell Invasion Assay

Transwell chambers (8 *μ*m pore size, Corning Inc., Lowell, MA, USA) precoated with Matrigel (BD Biosciences, Franklin Lakes, NJ, USA) that contained extracellular matrix proteins were used to assess cell invasion ability following the manufacturer's protocol. Briefly, MDA-MB-231 and HCC 1937 cells (5 × 10^4^) were suspended in 200 *μ*l serum-free DMEM and seeded into the upper chamber. DMEM containing 10% FBS was added to the lower chamber to serve as a chemoattractant. After incubation for 24 h at 37°C, noninvading cells on the upper surface of the filter were removed and cells that migrated to the lower surface were fixed, stained with 1% crystal violet. For quantification of cell invasion, 5 fields in each well were randomly selected and cells were counted by a light microscope at ×200 magnification.

### 2.9. Quantitative Reverse Transcription-Polymerase Chain Reaction

Total RNA of tissues or cells was extracted using TRIzol reagent (Invitrogen, Carlsbad, CA, USA) according to the manufacturer's instructions and it was converted into cDNA by PrimeScript™ RT-PCR kit according to the manufacturer's instructions (Takara, Tokyo, Japan). After the reverse transcription (RT) process, quantitative reverse-transcription polymerase chain (qRT-PCR) reaction was performed under the instruction of SYBR Green PCR Master Mix on a 7900HT Fast RT-PCR instrument (Applied Biosystems, Singapore). The amplification procedure was as follows: 95°C for 3 min, then 40 cycles at 95°C for 3 s, 60°C for 30°s and followed by 95°C for 15 s, 60°C for 15 s, and 95°C for 15 s. The relative expression of miRNA and that of mRNA were evaluated by threshold cycle (CT) value using the 2^−∆∆Ct^ method. The U6 or *β*-actin was used as controls, respectively. Primer sequences are provided in Table 1.

### 2.10. Western Blot Assay

After 48–72 h transfection, MDA-MB-231 and HCC 1937 cells were harvested and lysed by RIPA lysis buffer (80 *μ*l/well, Beyotime, Shanghai, China). BCA Protein Assay Kit (Beyotime, Jiangsu, China) was then used to quantify the protein concentrations. Equal amount of proteins were separated with sodium dodecyl sulfate-polycylamide gel electrophoresis (SDS-PAGE) and then transferred to 0.45-*μ*m nitrocellulose membranes (Beyotime) using the cold transfer buffer. The membranes were blocked in PBST (Shanghai Engineering Co.) containing 5% BSA for phospso-specific antibodies and PBST with 5% nonfat milk for other primary antibodies at room temperature for 1 h, and subsequently incubated with primary antibodies overnight at 4°C. After that, the membranes were washed with PBST and incubated with IRDye 680 donkey antimouse IgG-(*H* + *L*) (1 : 1,000 dilution; cat. no. 926-68072) or goat antirabbit.

IRDye 800CW secondary antibody (1 : 1,000 dilution; cat. no. 926-32211; LI-COR Biosciences, Lincoln, NE, USA) for 1 h. Immunereactive protein bands were detected with an Odyssey Scanning system (LI-COR Biosciences, Lincoln, NE, USA). The target bands' intensities were calculated and normalized to *β*-actin in order to determine relative protein concentration.

Antibodies used were list as follows: Anti-ASPP2 (1 : 20,000 dilu-tion; cat. no. ab181377; Abcam, Cambridge, UK), anti-*β*-actin (1 : 2,000 dilution; cat. no. sc-47778; Santa Cruz Biotechnology, Inc., Dallas, TX, USA), anti-Caspase-9 (1 : 1,000 dilution; cat. no. ab202068; Abcam), anti-Caspase-3 (1 : 1,000 dilution; cat. no. 9662; Cell Signaling Technology, Inc., Danvers, MA, USA), anti-PARP (1 : 1,000 dilution; cat. no. ab191217; Abcam), anti-Bcl2 (1 : 1000 dilution, cat. no. 4223, Cell Signaling Technology), anti-N-cadherin (1 : 2,000 dilution; cat. no. ab18203), anti-ZEB1 (1 : 1,000 dilution; cat. no. ab155249), anti-MMP2 (1 : 2,000 dilution; cat. no. ab37150), anti-AKT (1 : 1,000 dilution; cat. no. 9272), anti-phosphorylated (p-)AKT (ser-473; 1 : 1,000 dilution; cat. no. 4060) (all from Cell Signaling Technology, Inc.).

### 2.11. Luciferase Reporter Assay

For luciferase reporter assay, 293T cells were cultured to 80% confluence in 48-well plates, and cotransfected with 40 ng of psiCHECK-2-ASPP2-3′ UTR or psiCHECK-2-ASPP2-MUT-3′ UTR vector, plus 10 pmol of miR-30b mimics or negative control using Lipofectamine 2000 (Invitrogen), according to the manufacturer's instructions. After 24 h, cells were collected and firefly and Renilla luciferase activities were measured by using a Dual Luciferase Assay (Promega, Madison, WI, USA). Firefly luciferase activity was normalized to Renilla luciferase activity, and the ratio of firefly/renilla was recorded.

### 2.12. Statistical Analysis

Data from at least three independent experiments are presented as the mean ± standard (mean ± SD). Student's *t*-test was used for comparisons. *p* value < 0.05 was considered significant. GraphPad Prism version 6.0 (GraphPad, San Diego, CA, USA) was used for all statistical analyses.

## 3. Results

### 3.1. MiR-30b-5p Is Aberrantly Upregulated in Breast Cancer Tissues and Cell Lines

**Figure 1:**
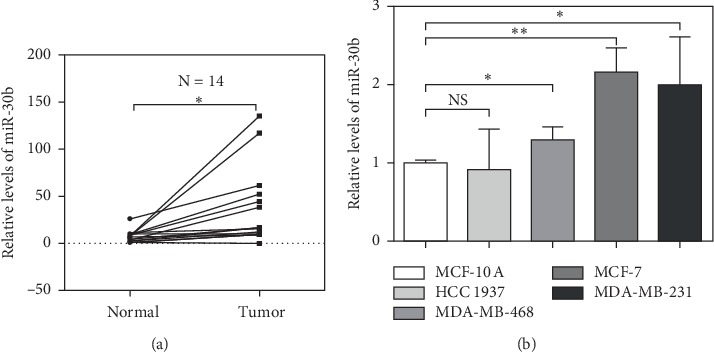
MiR-30b-5p is significantly upregulated in breast cancer specimens and cell lines. (a) The relative mRNA expression of miR-30b-5p in breast cancer tissues. (b) The relative mRNA level of miR-30b-5p in breast cancer cell lines. Data are represented as mean ± SD. ^*∗*^*p* < 0.05, ^*∗∗*^*p* < 0.01, ^*∗∗∗*^*p* < 0.001.

We found that the miR-30b-5p expression significantly increased in breast cancer tissues compared to adjacent noncancer tissues (Figure 1(a)). In addition, we determined the expression of miR-30b-5p in MDA-MB-231, HCC 1937, MCF-7, and MDA-MB-468 cells, in comparison with MCF-10A, a normal breast epithelial cell line. The result showed that miR-30b-5p expression was up-regulated in majority of breast cancer cell lines (MDA-MB-231, MCF-7, MDA-MB-468) compared to than MCF-10A cells (Figure 1(b)). These results indicated that expression of miR-30b-5p was upregulated in breast cancer.

### 3.2. MiR-30b-5p Promotes Cell Proliferation of MDA-MB-231 and HCC 1937 Cells

**Figure 2:**
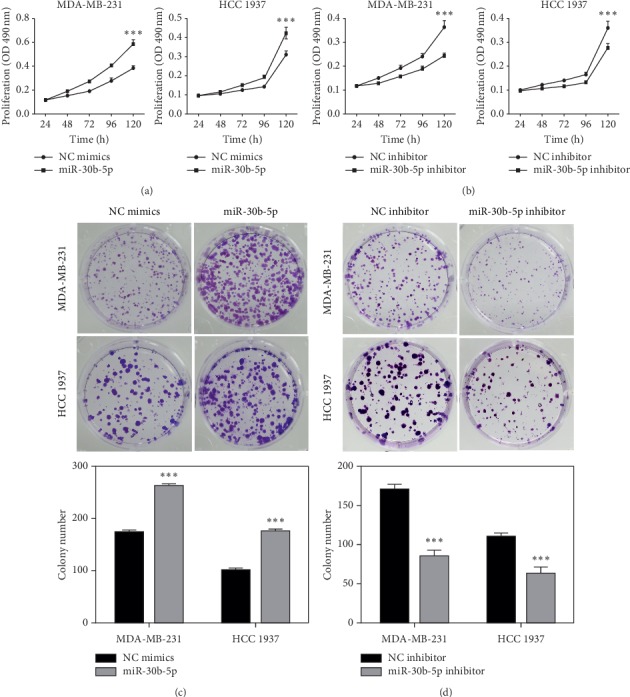
MiR-30b-5p promotes proliferation of breast cancer cells. (a) MTT assay showed miR-30b-5p promoted cell growth of MDA-MB-231 and HCC 1937 cells. (b) MTT assay showed miR-30b-5p inhibitor inhibited cell growth of MDA-MB-231 and HCC 1937 cells. (c) MiR-30b-5p promoted colony formation of MDA-MB-231 and HCC 1937 cells. (d) MiR-30b-5p inhibitor reduced colony formation of MDA-MB-231 and HCC 1937 cells. Data was represented as mean ± SD. ^*∗*^*p* < 0.05, ^*∗∗*^*p* < 0.01, ^*∗∗∗*^*p* < 0.001.

To reveal the role of miR-30b-5p in TNBC, MDA-MB-231, and HCC 1937 cells were transfected with miR-30b-5p mimics, miR-30b-5p inhibitors, and their corresponding NCs. Subsequently, MTT assay and colony formation assay were performed. The results showed that gain expression of miR-30b-5p increased the proliferation ability of MDA-MB-231 and HCC 1937 cells, whereas loss expression of miR-30b-5p decreased the proliferation ability of these two cell lines (Figures 2(a) and 2(b)). Similarly, colony formation assays further demonstrated that overexpression of miR-30b-5p promoted the colony formation ability of MDA-MB-231 and HCC 1937 cells compared with that in control cells (Figure 2(c)), while downregulation of miR-30b-5p presented a decrease in the colony formation ability (Figure 2(d)). Collectively, these results indicated that miR-30b-5p played an important role in the proliferation of TNBC cells.

### 3.3. MiR-30b-5p Reduces Cell Apoptosis of MDA-MB-231 and HCC 1937 Cells

**Figure 3:**
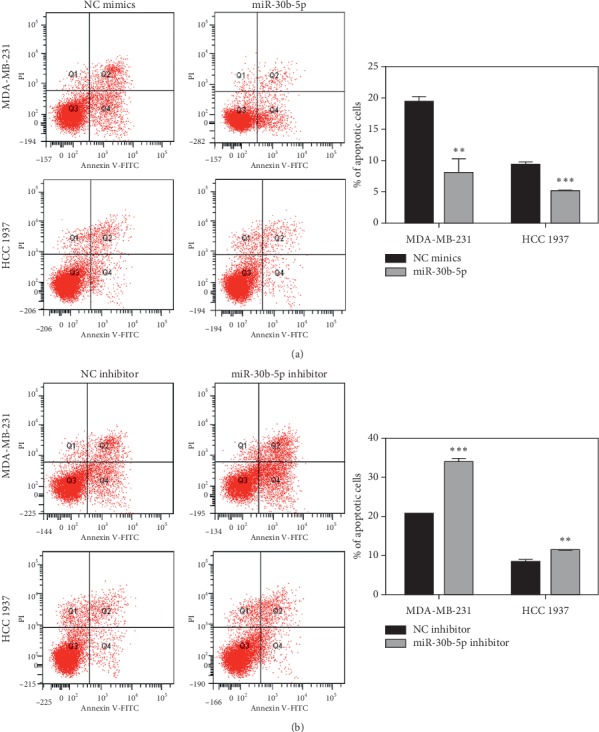
MiR-30b-5p reduces cell apoptosis of breast cancer cells. (a) Overexpression of miR-30b-5p suppressed cell apoptosis of MDA-MB-231 and HCC 1937 cells. (b) Inhibition of miR-30b-5p increased cell apoptosis of MDA-MB-231 and HCC 1937 cells. Data are represented as mean ± SD. ^*∗*^*p* < 0.05, ^*∗∗*^*p* < 0.01, ^*∗∗∗*^*p* < 0.001.

The functional influence of miR-30b-5p on the apoptosis was analyzed using flow cytometry. Compared with the control, overexpression of miR-30b-5p led to a significant reduction in the percentage of cells undergoing apoptosis both in MDA-MB-231 and HCC 1937 cells (Figure 3(a)). In contrast, miR-30b-5p inhibitor noticeably increased cell apoptosis in MDA-MB-231 and HCC 1937 cells (Figure 3(b)). Accordingly, we demonstrated that upregulation of miR-30b-5p could increase the inhibition of apoptosis in TNBC cell lines.

### 3.4. MiR-30b-5p Improves Cell Migration and Invasion Abilities of MDA-MB-231 and HCC 1937 Cells

**Figure 4:**
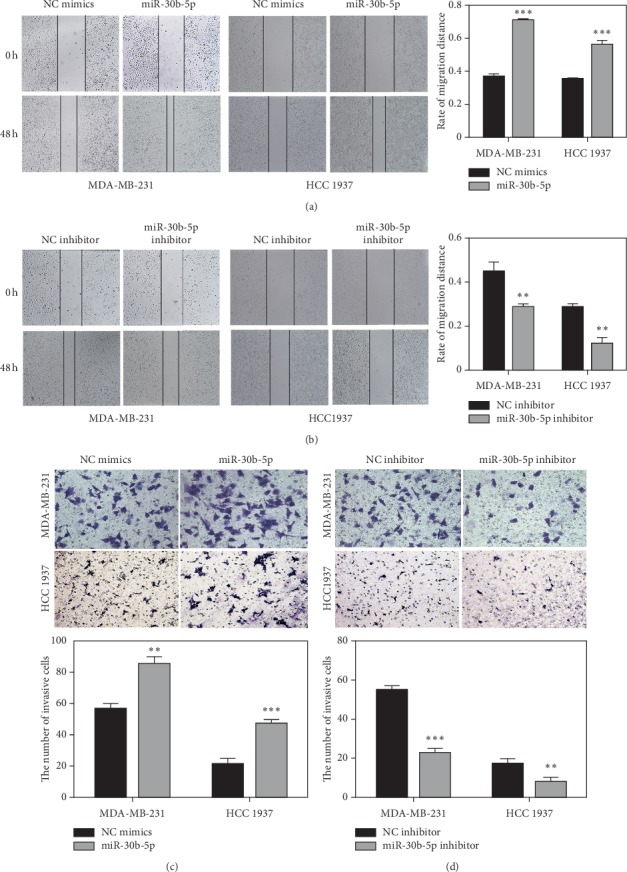
MiR-30b-5p promotes motility of breast cancer cells. (a) Wound healing assay indicated that miR-30b-5p increased migration of MDA-MB-231 and HCC 1937 cells. (b) Wound healing assay indicated that miR-30b-5p inhibitor inhibited migration of MDA-MB-231 and HCC 1937 cells. (c) Transwell assay showed that miR-30b-5p promoted MDA-MB-231 and HCC 1937 cells invasion. (d) Transwell assay showed that miR-30b-5p inhibitor decreased MDA-MB-231 and HCC 1937 cells invasion. Data are represented as mean ± SD. ^*∗*^*p* < 0.05, ^*∗∗*^*p* < 0.01, ^*∗∗∗*^*p* < 0.001.

To examine the role of miR-30b-5p in the metastatic processes of TNBC, the wound healing assay and transwell assay were performed in MDA-MB-231 and HCC 1937 cells. As shown in Figure 4(a), the migration rate was increased in the wound of cells transfected with miR-30b-5p mimics when compared with that in control group. On the contrary, the rate of wound closure was decreased in the wound of cells transfected with miR-30b-5p inhibitors and migrated slower than the control group (Figure 4(b)). Additionally, transwell assay indicated that upregulation of miR-30b-5p could increase the cell invasion in both MDA-MB-231 and HCC 1937 cells (Figure 4(c)). As expected, cells transfected with miR-30b-5p inhibitor displayed the opposite effects (Figure 4(d)). Taken together, the data indicated that miR-30b-5p indeed promoted the migration and invasion abilities of TNBC cells.

### 3.5. MiR-30b-5p Regulates Expression of Genes Involved in Cell Apoptosis and Invasion of MDA-MB-231 and HCC 1937 Cells

**Figure 5:**
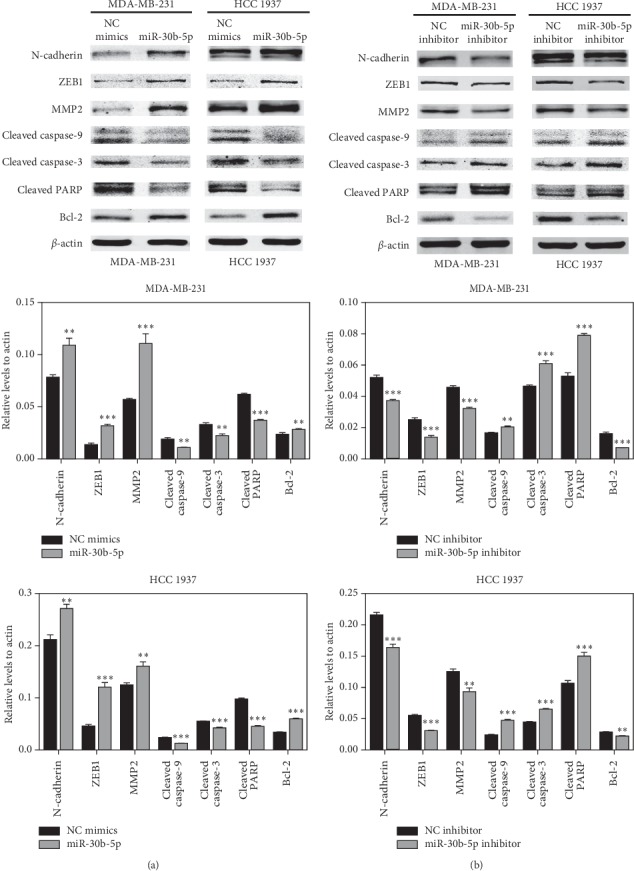
Effects of miR-30b-5p on the expression of apoptosis and EMT-related genes. (a) The expressions of apoptosis and EMT related proteins in MDA-MB-231 and HCC 1937 cells transfected with miR-30b-5p or NC mimics, respectively. (b) The expressions of apoptosis and EMT related proteins in MDA-MB-231 and HCC 1937 cells transfected with miR-30b-5p inhibitor or NC inhibitor, respectively. Data was represented as mean ± SD. ^*∗*^*p* < 0.05, ^*∗∗*^*p* < 0.01, ^*∗∗∗*^*p* < 0.001.

To further clarify the mechanisms of miR-30b-5p in regulating the biological function of MDA-MB-231 and HCC 1937 cells, we carried out Western blot assay for candidate downstream genes responsible for apoptosis and EMT, in the MDA-MB-231 and HCC 1937 cells transfected with miR-30b-5p mimics or inhibitors, and their responding NCs. As shown in Figure 5(a), the results showed that upregulation of miR-30b-5p elevated the protein levels of N-cadherin, ZEB1, and MMP2. Besides, the momentous effect of miR-30b-5p on apoptosis was verified, as decreased activating cleavage of PARP, Caspase-3, and Caspase-9 was found in TNBC cells that with miR-30b-5p overexpression. Nevertheless, mir-30b-5p inhibitor played an opposite role (Figure 5(b)). These results further confirmed that miR-30b-5p play an important role in the apoptosis and metastasis of MDA-MB-231 and HCC 1937 cells.

### 3.6. ASPP2 is a Direct Target Gene of MiR-30b-5p

**Figure 6:**
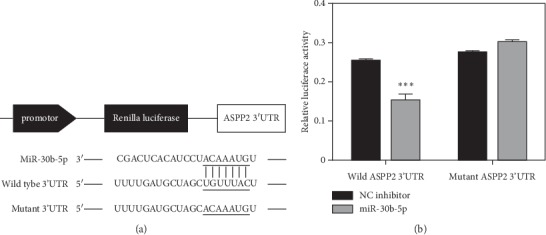
ASPP2 is a target gene of miR-30b-5p. (a) Binding sites of miR-30b-5p and ASPP2 3′-UTR. (b) MiR-30b-5p decreased luciferase activity in wild-type group. Data was represented as mean ± SD. ^*∗*^*p* < 0.05, ^*∗∗*^*p* < 0.01, ^*∗∗∗*^*p* < 0.001.

On the basis of miRNA target analysis algorithms (microRNA.org and TargetScan), ASPP2 is a potential target mRNA of miR-30b (Figure 6(a)). In addition, ASPP2 plays important roles in stimulating cell apoptosis and inhibiting epithelial-mesenchymal Transition (EMT) process in breast cancer [17,18]. Therefore, ASPP2 was selected for further analysis. We performed luciferase reporter assay and the result showed that the overexpression of miR-30b-5p significantly inhibited the firefly luciferase reporter activity of the wild-type ASPP2 3′-UTR (Figure 6(b)). The data suggested that ASPP2 is a direct target of miR-30b-5p.

**Figure 7:**
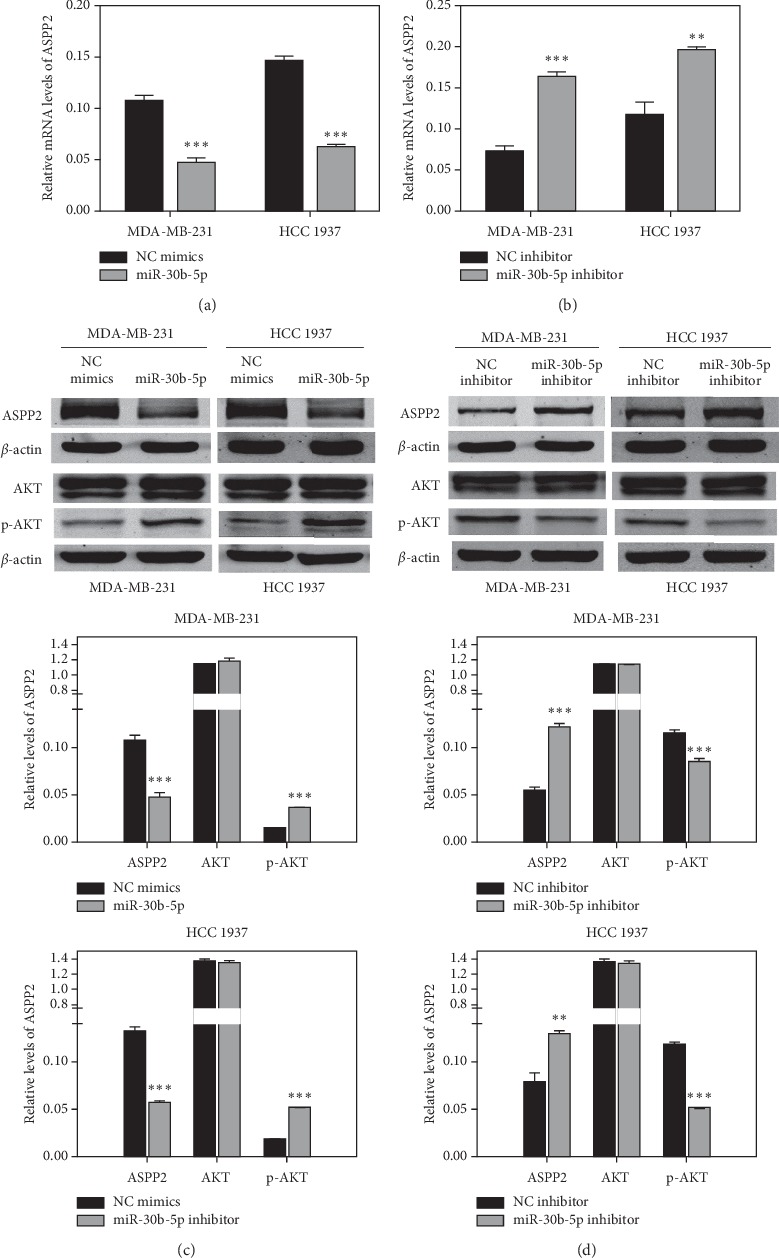
MiR-30b-5p regulates the expression of ASPP2/AKT axis. (a) MiR-30b-5p suppressed mRNA expression of ASPP2 in MDA-MB-231 and HCC 1937 cells. (b) MiR-30b-5p inhibitor increased mRNA expression of ASPP2. (c) MiR-30b-5p suppressed protein expression of ASPP2 but contributed to the activation of p-AKT in MDA-MB-231 and HCC 1937 cells. (d) MiR-30b-5p inhibitor increased protein expression of ASPP2 and prevented the activation of p-AKT. Data are represented as mean ± SD. ^*∗*^*p* < 0.05, ^*∗∗*^*p* < 0.01, ^*∗∗∗*^*p* < 0.001.

To further confirm the direct targeted link between miR-30b-5p and ASPP2. We observed that both mRNA and protein levels of ASPP2 were significantly reduced after miR-30b-5p overexpression (Figures 7(a) and 7(c)), and conversely, the mRNA and protein levels of ASPP2 were increased by transfection of miR-30b-5p inhibitor in both MDA-MB-231 and HCC 1937 cells (Figures 7(b) and 7(d)). Our prophase research found that as ASPP2 siRNA inhibited the expression of ASPP2, the expression of p-AKT increased [19]. In present study, we also found that miR-30b-5p transfection significantly promoted the expression of p-AKT, whereas miR-30b-5p inhibitor resulted in the reduction of p-AKT expression (Figures 7(c) and 7(d)). The above results indicated that miR-30b-5p may directly target to the ASPP2, inhibit its expression and lead to the p-AKT activation.

## 4. Discussion

Triple negative breast cancer (TNBC) has been considered as one of the worst prognosis of all breast cancer subtypes, but the novel biomarkers and targeted treatments of TNBC are scarce, [20]. A growing body of evidence indicate that miRNAs are involved in the regulation of specific genes, contributing to vary levels of control over tumorigenesis, metastasis and drug resistance [9,21,22]. In TNBC, numerous miRNAs have been regarded to serve as the potential targets for the anticancer therapy [23]. For instance, overexpression of miR-200b and miR-429-5p markedly inhibit cell growth, migration and invasiveness of TNBC cells [24]. Restoration of miR-34a in partial TNBC cells can activate senescence and promote sensitivity to dasatinib, which is dependent on the suppression of proto-oncogene c-SRC [25]. The previous study has investigated that upregulation of miR-30b was observed in aggressive carcinoma of the oral tongue in young patients [25]. In human melanoma, miR-30b overexpression corresponded with increased metastasis, tumor thickness and advancing stage (I to III), it also plays a crucial role on tumor cell invasion and immune modulation, predominantly by suppressing GALNT7 [26]. It is worth noting that miR-30b can significantly reduce cell apoptosis via inhibiting Sema3A and p38MAPK, contributing to the axon outgrowth of retinal ganglion cells [27]. However, miR-30b has been reported to serve as a tumor suppressor in cell proliferation and metastasis in renal cell carcinoma [28]. These findings indicate that miR-30b-5p is likely to play different even opposite roles in different tumors dependent on its tissue specificity. In this research, our results demonstrated that miR-30b-5p was frequently up-regulated in breast cancer tissues and cell lines, which was consistent with a previous microRNA profiling revealing that overexpression of miR-30b-5p was detected in both the blood and tissue samples of breast cancer patients [29]. Moreover, we found that upregulation of miR-30b-5p promoted cell growth, migration, invasiveness, and reduced cell apoptosis in TNBC (MDA-MB-231 and HCC 1937) cells, whereas inhibition of miR-30b-5p had an opposite effect. These findings indicate that miR-30b-5p serves as an oncogene in TNBC proliferation and metastasis, enriching the tumor promoting function of miR-30b-5p in various types of tumors. Notably, miR-30b-5p alone or in combination with trastuzumab reduces the cell proliferation in Her2+ breast cancer cells. But in breast cancer MDA-MB-453 cells, miR-30b-5p is upregulated by proteasome inhibitor PS-341 treatment and able to promote cell proliferation and antiapoptosis [30,31]. It is found that the expression of miR-30b-5p is downregulated by Her2, thereby inhibiting cell growth in proliferative lupus nephritis (LN) [32]. Therefore, we suppose that the discrepant effects in Her2+ breast cancers and TNBC may at least in part be dependent on the Her2 status, while the concrete regulator mechanism remains require further investigation.

ASPP2 (apoptosis-stimulating p53-binding protein 2), encoded by TP53BP2 gene, is considered to play a crucial role on apoptosis genesis via binding to p53 family and enhancing their transcriptional activities towards pro-apoptosis gene [33–35]. Interestingly, in addition to promoting cell apoptosis via binding to p53, ASPP2 has recently been confirmed to function in p53-independent manners and the aberrant expression of ASPP2 is involved in numerous cellular functions in tumors, not only cell apoptosis but also cell metastasis, senescence, and autophagy [18,36–39]. Similarly, it has been reported that ASPP2 is downregulated in majority of human tumors, such as hepatocellular carcinoma, pancreatic cancer, and breast cancer [40–42]. N terminus of ASPP2 is identified as a potential Ras-binding (RB)/Ras-association (RA) domain, contributing to the binding and collaborating between ASPP2 and Ras [43]. ASPP2 can potentiate Ras signaling and even convert its oncogenic role to tumor-suppressive role on cell apoptosis and senescence [44,45]. In gastric cancer, ASPP2 inhibited ubiquitin-dependent degradation of Smad7 by interacting with ITCH, leading to the suppression of TGF-*β*1-Smad2/3 signaling, which reduced migration and invasion of gastric cancer cells both in vitro and in vivo [37]. Currently, ASPP2 as a tumor suppressor involved in the cell apoptosis and EMT process in breast cancer has attracted much more attention [18,46].

In our experiments, we found ASPP2 might be a potential target gene of miR-30b-5p. To validate the link between miR-30b-5p and ASPP2, luciferase reporter assay was performed, further results demonstrating that miR-30b-5p could directly target ASPP2. Besides, the expression of ASPP2 mRNA and protein levels was significantly downregulated when cells were transfected with miR-30b-5p mimics, and its expression could be reversed by miR-30b-5p inhibitors. Next, to explore the molecular mechanism induced by miR-30b-5p, we investigated its impact on the levels of a battery of apoptosis and EMT related genes and found that miR-30b-5p could promote the expression of N-cadherin, ZEB1, MMP2, and Bcl2 protein while inhibiting the expression of Cleaved caspase-9, Cleaved caspase-3 and Cleaved PARP protein in TNBC cells. These results further confirmed that miR-30b-5p reduced cell apoptosis and promoted EMT process, which was consistent with the effects of ASPP2 silencing [18,46]. In addition, the activation of p-AKT was found to be induced by miR-30b-5p overexpression in TNBC cells. The activation of p-AKT is an important antiapoptotic factor via inhibiting the downstream genes, including Caspase9, Bad, P21 [47]. Besides, it is also correlated with EMT in endometrial carcinoma cells [48]. Collectively, we demonstrated that miR-30b-5p may suppress cell apoptosis and promote EMT through the miR-30b-5p/ASPP2/AKT axis.

## 5. Conclusion

Our study found that miR-30b-5p was upregulated in breast cancer. Overexpression of miR-30b-5p could reduce the cell apoptosis while contributing to the cell proliferation, migration, and invasion in TNBC, and its oncogenic effects at least partly through mediating its downstream target gene ASPP2, which is a critical regulator of cell apoptosis and EMT process of TNBC by activating p-AKT expression. This study identified the miR-30b-5p/ASPP2/AKT axis plays an important role in TNBC and may provide new insights into the therapeutic target for TNBC clinical treatment.

## Figures and Tables

**Table 1:** Nucleotide sequences of primers used for PCR.

Gene	Primer	Sequence(5′-3′)
ASPP2	Forward	CTGTGCAAAGAACCCGGCG
	Reverse	CAACTGGACGTTCAGAGCCACA

*β*-actin	Forward	CAGAGCCTCGCCTTTGCC
	Reverse	GTCGCCCACATAGGAATC

miR-30b-5p	Forward	TGTAAACATCCTACACTCAGCT
	Reverse	CAGTGCGTGTCGTGGAGT

U6	Forward	CTCGCTTCGGCAGCACA
	Reverse	AACGCTTCACGAATTTGCGT

## Data Availability

The experimental data used to support the findings of this study are included within the article.

## Supplementary Materials

Supplementary Materials Supplement Table I: patient clinical pathological characteristics (summary).
