# Supplementary material for: Mir-30b-5p Promotes Proliferation, Migration, and Invasion of Breast Cancer Cells via Targeting ASPP2
Source: Biomed Res Int. 2020 Apr 29;2020:7907269. doi: 10.1155/2020/7907269 (PMC7210518; doi:10.1155/2020/7907269)
Supplement: Supplementary Materials — Supplement Table I: patient clinical pathological characteristics (summary). [file 7907269.f1.pdf]

Supplement Table I. Patient clinicalpathological characteristics (Summary).

Characteristics	Patient Number	miR-30b-5p Expression level					
		Low		No difference		High	
		No.	%	No.	%	No.	%
Age							
⩽40	1	0	0	0	0	1	100
40-60	9	1	11.11	2	22.22	6	66.67
>60	4	0	0	0	0	4	100
Clinical stage at diagnosis							
I	3	1	33.33	1	33.33	1	33.33
II	8	0	0	0	0	8	0
III	3	0	0	1	33.33	2	66.67
Tumor stage (size cm)							
T1(≤2.0)	5	1	20	1	20	3	60
T2(>2.0 to ≤5.0)	7	0	0	1	14.29	6	85.71
T3（>5.0）	2	0	0	0	0	2	100
Tumor number							
Single	10	1	10	2	20	7	70
Multiple	4	0	0	0	0	4	100
Nodal stage							
N0 (node negative)	8	1	12.5	1	12.5	6	75
N1 (1-3 positive nodes)	3	0	0	1	33.33	2	66.67
N2 (4-9 positive nodes)	2	0	0	0	0	2	100
N3 (≥10 positive nodes)	1	0	0	0	0	1	100
Histological grade							
Grade 1	2	1	50	0	0	1	50
Grade 2	5	0	0	1	20	4	80
Grade 3	7	0	0	1	14.29	6	85.71
Subtypes of breast cancer							
Luminal A-like	2	1	50	0	0	1	50
Luminal B-like	3	0	0	1	33.33	2	66.67
HER2 positive	4	0	0	1	25	3	75
Triple negative	5	0	0	0	0	5	100
Not known	0	0	0	0	0	0	0
